# Fluorescence Bronchoscopic Surveillance in Patients With a History of Non-Small Cell Lung Cancer

**DOI:** 10.1155/DTE.6.1

**Published:** 1999

**Authors:** Tracey Lee Weigel, Pamela J. Kosco, Sanja Dacic, Samuel Yousem, James D. Luketich

**Affiliations:** 1 Department of Surgery Section of Thoracic Surgery University of Pittsburgh Medical Center C800, 200 Lothrop Street Pittsburgh PA 15213 USA; 2 Department of Pathology Section of Thoracic Surgery University of Pittsburgh Medical Center C800, 200 Lothrop Street Pittsburgh PA 15213 USA

## Abstract

*Background* Second lung primaries occur at a rate of 2% per patient per year after curative resection for non-small cell lung carcinoma (NSCLC). The aim of this study was to evaluate the role of fluorescence bronchoscopy using the Xillix^®^ LIFE-Lung Fluorescent Endoscopy System^TM^ (LIFE-Lung system) in the surveillance of patients for second NSCLC primaries after resection or curative photodynamic therapy (PDT).

*Methods* NSCLC patients who were disease-free following resection or endobronchial PDT were identified and recruited to participate in a LIFE bronchoscopy surveillance program. All suspicious areas were biopsied; areas of apparent normal mucosa served as negative controls. Biopsy specimens were reviewed by a single pulmonary pathologist.

*Results* Thirty-six patients underwent 53 surveillance LIFE bronchoscopies and 6/112 biopsies revealed intraepithelial neoplasia (IEN) or invasive carcinoma in 6/36 (17%) of patients. The overall relative sensitivity of LIFE versus conventional bronchoscopy was 165% with a negative predictive value of 0.96, for the post-resection subset of patients these values increased to 200% and 0.97, respectively.

*Conclusions* Surveillance LIFE bronchoscopy identified intraepithelial or invasive lesions in 17% of patients previously thought to be disease-free. These data support future multicenter trials on fluorescence bronchoscopic surveillance of NSCLC patients after curative surgical resection or PDT.


*Diagnostic and Therapeutic Endoscopy*, Vol. 6, pp. 1–7
Reprints available directly from the publisher
Photocopying permitted by license only

© 1999 OPA (Overseas Publishers Association) N.V.
Published by license under
the Harwood Academic Publishers imprint,
part of The Gordon and Breach Publishing Group.
Printed in Malaysia.

### Fluorescence Bronchoscopic Surveillance in Patients with a History of Non-Small Cell Lung Cancer

TRACEY LEE WEIGEL[a,*], PAMELA J. KOSCO[a], SANJA DACIC[b], SAMUEL YOUSEM[b] and JAMES D. LUKETICH[a]

[a]*Department of Surgery,* [b]*Department of Pathology, Section of Thoracic Surgery, University of Pittsburgh Medical Center, C800, 200 Lothrop Street, Pittsburgh, PA 15213, USA*

*(Received 29 October 1998; Revised 29 June 1999; In final form 6 July 1999)*

***Background*** **Second lung primaries occur at a rate of 2% per patient per year after curative resection for non-small cell lung carcinoma (NSCLC). The aim of this study was to evaluate the role of fluorescence bronchoscopy using the Xillix® LIFE-Lung Fluorescent Endoscopy System™ (LIFE-Lung system) in the surveillance of patients for second NSCLC primaries after resection or curative photodynamic therapy (PDT).**

***Methods*** **NSCLC patients who were disease-free following resection or endobronchial PDT were identified and recruited to participate in a LIFE bronchoscopy surveillance program. All suspicious areas were biopsied; areas of apparent normal mucosa served as negative controls. Biopsy specimens were reviewed by a single pulmonary pathologist.**

***Results*** **Thirty-six patients underwent 53 surveillance LIFE bronchoscopies and 6/112 biopsies revealed intraepithelial neoplasia (IEN) or invasive carcinoma in 6/36 (17%) of patients. The overall relative sensitivity of LIFE versus conventional bronchoscopy was 165% with a negative predictive value of 0.96, for the post-resection subset of patients these values increased to 200% and 0.97, respectively.**

***Conclusions*** **Surveillance LIFE bronchoscopy identified intraepithelial or invasive lesions in 17% of patients previously thought to be disease-free. These data support future multicenter trials on fluorescence bronchoscopic surveillance of NSCLC patients after curative surgical resection or PDT.**

*Keywords:* Fluorescent bronchoscopy, Intraepithelial neoplasia, Non-small cell lung cancer, Surveillance

### INTRODUCTION

Only about 14% of patients with invasive lung cancer can be cured using conventional treatment modalities (surgery, chemotherapy and/or radiation therapy) [1]. By the time a carcinoma of the lung produces symptoms and is diagnosed, it has usually progressed locally beyond resectability (Stage IIIB)

* Corresponding author. Tel.: +1-412-647-7555. Fax: +1-412-647-7550.

or metastasized (Stage IV). At present, only one out of eight people diagnosed with non-small cell lung carcinoma (NSCLC) is alive at five years. The only way to significantly impact on the high mortality from lung cancer appears to be identification of the disease at an earlier stage, prior to its becoming a systemic disease.

The histological response of the respiratory mucosa to exogenous environmental stresses is a predictable progression from normal mucosa through metaplasia, dysplasia, carcinoma *in situ* (CIS) and eventually resulting in invasive carcinoma [2]. On an average, a period of four to five years exists during which time individuals exfoliate markedly atypical cells into bronchial secretions that antedates the progression to an invasive tracheobronchial carcinoma [3]. During this exfoliative period, the tracheobronchial trees of these individuals may harbor occult intraepithelial neoplasias (IENs) below the resolution of conventional bronchoscopy but potentially detectable by fluorescent bronchoscopy.

Currently, lung carcinomas in high-risk patients are most commonly identified through periodic sputum cytologic analysis and/or standard chest X-rays. Both of these methodologies are less than optimum screening modalities; the former does not localize the lesion within the tracheobronchial tree and the latter is neither sensitive nor specific for the malignancy.

Conventional white light bronchoscopy (WLB) is thought by many to be a useful tool in localizing radiographically occult NSCLCs in the tracheobronchial tree. In actuality, only 29% of CISs and 69% of microinvasive tumors are identified endoscopically with WLB even by experienced bronchoscopists [4]. Early NSCLCs and even IENs are detectable with a highly sensitive, relatively new method of bronchoscopy that utilizes a blue light to induce autofluorescence of the bronchial surface; the Xillix® LIFE-Lung Fluorescent Endoscopy System™.

In 1996 the LIFE-Lung system, an autofluorescent tracheobronchial imaging system developed by the British Columbia Cancer Research Centre in conjunction with Xillix Technologies, was approved by the Food and Drug Administration (FDA). LIFE-Lung utilizes a helium–cadmium laser to generate monochromatic blue (442 nm) light and induce autofluorescence of the bronchial mucosa, capitalizing on the fact that the normal and abnormal tissues have different patterns of autofluorescence. *In vivo* spectroscopy, with an optical multichannel analyzer, enables the LIFE-Lung system to identify dysplasia, CIS and microinvasive carcinomas. The different autofluorescence patterns characteristic of IENs and early, invasive carcinomas are detectable by the LIFE-Lung system in the absence of exogenous photosensitizers [5].

The LIFE-Lung system is comprised of a 125 mW helium–cadmium laser as a source of blue light (442 nm), two image-intensified CCD cameras with red and green filters, computer and a color video monitor [6]. Blue light is delivered to the bronchial surface via a standard, Olympus BF20 bronchoscope. Non-collimated light (12–15 mW) is emitted from the distal end of the bronchoscope. Tracheobronchial mucosal and submucosal fluorescence in both the red (> 630 nm) and the green (520 nm) spectra are simultaneously captured, integrated and displayed in near real time on a color video monitor.

In a normal bronchus, the predominant source of fluorescence is the submucosa [6]. Several theories exist as to the etiology of the diminished autofluorescence of areas of dysplasia, CIS and invasive carcinoma. Morphologic properties may explain these differences in autofluorescence, i.e. they may merely be a consequence of the thickening of the abnormal epithelium or an increase in the bronchial capillary density of IENs and invasive carcinomas. Alternatively, a loss of natural fluorophors as lesions progress from normal to malignant phenotype may be responsible for the differences in their autofluorescence properties.

The aim of the current IRB approved LIFE Bronchoscopy Surveillance Program for NSCLC patients at the University of Pittsburgh Medical Center is to determine the efficacy of the LIFE-Lung imaging system in the surveillance of both post-resection patients and photodynamic therapy (PDT) patients treated with curative intent.

The largest subset of patients, those with NSCLC who have undergone a prior complete surgical resection and remain disease-free, are identified from the Summit® database used by the University of Pittsburgh, Section of Thoracic Surgery. This database is collected prospectively and includes all patients treated by the Section of Thoracic Surgery at the University of Pittsburgh. Post-resection patients are contacted and their disease status verified. If eligible, they are offered a surveillance LIFE bronchoscopic exam.

The second population of patients offered LIFE surveillance at the University of Pittsburgh were those with early and/or proximal NSCLCs (T0–1N0M0) treated curatively with PDT identified from a separate, prospectively collected database. LIFE-bronchoscopy was performed $\sim$6 weeks post-tracheobronchial PDT treatment to re-evaluate the treated mucosa and to guide biopsies of any suspicious areas that might represent persistent and/or recurrent disease. Biopsies were performed at all sites previously treated with PDT, even if the mucosa appeared normal, to confirm complete histologic eradication of all lesions.

The standard interval between PDT treatments with curative intent at our institution is between 4 and 6 weeks. We opted to perform a LIFE exam at that time, recognizing that the specificity of the LIFE bronchoscopic exam post-PDT might be lower than previously reported by Lam *et al.* [7] due to the retained Photofrin II™. We accepted the potentially higher number of false positives detected by LIFE because delaying the exam beyond 6 weeks post-PDT (to allow for further clearance of Photofrin II) could compromise patient care if additional PDT was required.

All surveillance LIFE bronchoscopic exams were conducted as outpatient procedures using intravenous conscious sedation. Aerosolized and viscous 1% lidocaine, as well as cetacaine spray, were employed to achieve topical anesthesia of the oro- and hypopharynx. Lidocaine (1%) was instilled on the vocal cords and intratracheally, as needed, to maintain adequate topical anesthesia during the exam. Patient sedation and amnesia were achieved with intravenous versed and fentanyl.

Conventional WLB was carried out first using an Olympus BF20D™ fiberoptic bronchoscope. The entire tracheobronchial tree was inspected, the exam was videotaped and images were taken of any abnormalities. Lesions were classified clinically into three categories according to the clinical classification system first described by Lam *et al.* [7]. On white light exam normal mucosa was classified as Class I; areas with non-specific erythema, swelling or thickening of the mucosa, trauma, anatomic anomalies or granulation tissue were classified as Class II; and areas or nodularity, mucosal irregularity or focal thickening were classified as Class III (Fig. 1(a)). On LIFE exam, bright green areas represent normal mucosa and were defined as Class I (Fig. 1(b) and Fig. 2); slightly brown lesions with ill-defined margins were classified as Class II and definite brown or reddish-brown lesions with discrete borders were designated as Class III (Fig. 3).

The exact locations of all Class II and Class III changes were recorded on video and in the image management program of the LIFE system to afford retrieval to facilitate future examinations. Fluorescence bronchoscopy was performed using the LIFE-Lung system following completion of the WLB by changing to the helium–cadmium light source and connecting the LIFE camera. Once LIFE bronchoscopy has begun revisions were not permitted to the classifications assigned to mucosal lesions during the preceding WLB. All Class II and Class III lesions were biopsied and random biopsies were obtained from visually (by both WLB and LIFE) normal mucosa.

PDT using Photofrin™ ($\sim$1.5 mg/kg) delivering an energy dose of 100–200 J/cm$^2$ over 250–500 s was performed on all areas of histologically-confirmed CIS or microinvasive cancer identified by either WLB or LIFE exam. A "clean-up" WLB was performed on post-operative day one following PDT to debride/remove obstructing, tumor-mucosal casts.

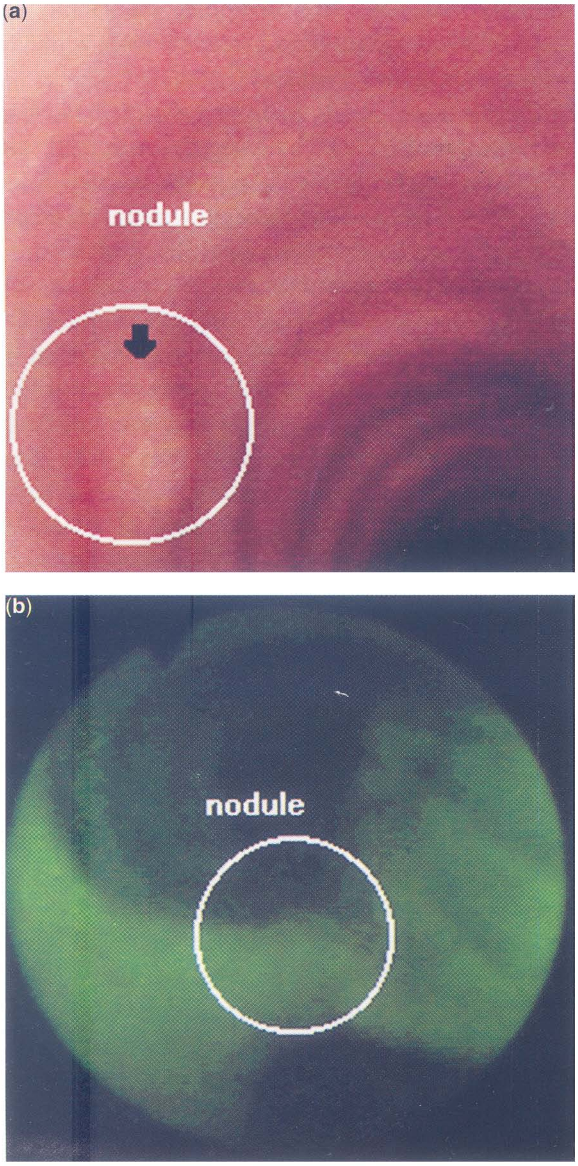

FIGURE 1 (a) Class II WLB image. (b) Corresponding Class I LIFE image of biopsy proven overlying benign submucosal nodule.

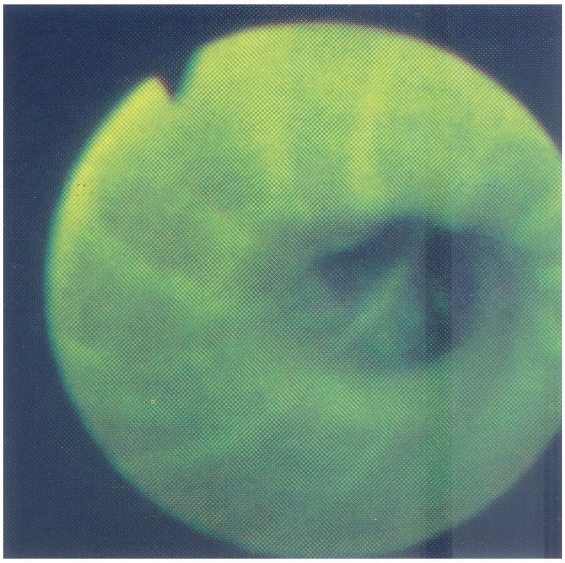

FIGURE 2 Normal LIFE exam image (Class I) of left upper lobe lingula showing bright green areas of normal mucosa.

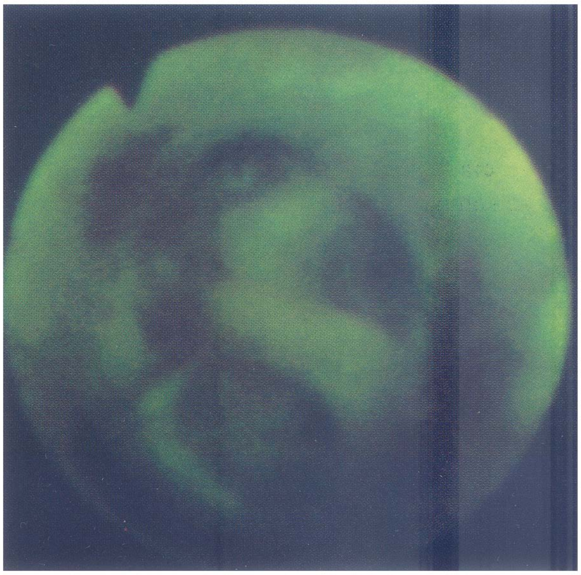

FIGURE 3 Class III LIFE image of right upper lobe showing definite reddish-brown areas with discrete borders.

CIS and microinvasive lesions treated with PDT were re-examined with both WLB and LIFE bronchoscopy at four months post-PDT to determine the durability of response. Additional PDT and/or surgical resection was performed if there was histologic evidence of recurrence or persistence of severe dysplasia and/or progression to an invasive carcinoma. All pathologic evaluations of the mucosal biopsies were made without knowledge of bronchoscopic findings.

### RESULTS

Over the twelve-month period from 12/97 to 12/98 a total of 36 patients underwent 53 surveillance LIFE exams (Table I). Thirty-four patients who were status-post-complete resection of a NSCLC underwent 46 surveillance LIFE exams. Of this 82% (28/34) of the patients had early stage disease (Stage I or II); the remaining six patients (18%) had Stage IIIA NSCLC. An IEN or invasive carcinoma was identified in 5/34 (15%) of the post-resection patients screened. The addition of the LIFE exam to conventional WLB increased the overall sensitivity of post-operative screening from 33% to 66%. In the post-surgical population of patients, the relative overall sensitivity of LIFE versus WLB was 200%. LIFE bronchoscopy correctly identified 2/3 (67%) of the IENs versus 0/3 (0%) for WLB. The negative predictive value of LIFE bronchoscopy in post-resection patients was 0.97 (Table II).

We performed 11 LIFE exams on five patients that were status-post-PDT with curative intent. Three of these patients also had a resection of a separate NSCLC primary. Eight lesions were identified in these five patients (one patient had four separate lesions) treated definitively with nine courses of PDT: two patients required one course, two patients required two courses and one patient required three courses to eradicate all histologic disease.

Only one lesion was detected in the post-PDT subset of patients and it was detected by both LIFE and WLB. No IENs were identified in the post-PDT subset of patients (Table III).

TABLE I Demographics and disease status post-curative resection or PDT ($n = 36$)

| | | | | |
|---|---|---|---|---|
| Gender | Male | 22 | Female | 14 |
| Age | Average | 65 | Range | 42–84 |
| Histology | NSCLC | 35 | SCLC | 1 |
| | Adenocarcinoma | 14 | | |
| | Squamous cell | 19 | | |
| | Poorly differentiated | 2 | | |
| Treatment | Surgery | 34 | PDT only | 2 |
| | Wedge resection | 3 | | |
| | Lobectomy | 24 | | |
| | Lingulectomy | 1 | | |
| | Pneumonectomy | 3 | | |
| | Surgery plus PDT | 3 | | |
| Stage (resection patients) | IA | 6 | | |
| | IB | 14 | | |
| | IIA | 4 | | |
| | IIB | 4 | | |
| | IIIA | 6 | | |

### DISCUSSION

In 1998 over 160,000 individuals died of lung cancer in the United States alone, exceeding the total mortality from breast, prostate, pancreatic and all lymphomas and leukemias. Much controversy exists with respect to the current practice and future standard of care with respect to screening for breast, colon and prostrate cancer [8]. "Virtually all organizations, however, concur that screening for lung cancer is not justified... the American College of

TABLE II Detection with LIFE versus WLB post-resection patients ($n = 89$ evaluable biopsies)*

| | WLB | LIFE |
|---|---|---|
| True positive | 2 | 4 |
| False negative | 4 | 2 |
| True negative | 72 | 40 |
| False positive | 11 | 43 |
| IEN + invasive carcinoma | | |
| Sensitivity | 0.33 | 0.66 |
| Specificity | 0.86 | 0.48 |
| IEN only | | |
| Sensitivity | 0.00 | 0.66 |
| Specificity | 0.86 | 0.48 |

*Results of a per lesion analysis.

TABLE III Detection with LIFE versus WLB Post-PDT patients ($n = 35$ evaluable biopsies)*

| | WLB | LIFE |
|---|---|---|
| True positive | 1 | 1 |
| False negative | 0 | 0 |
| True negative | 33 | 9 |
| False positive | 1 | 25 |
| IEN + invasive carcinoma | | |
| Sensitivity | 1.0 | 1.0 |
| Specificity | 0.97 | 0.26 |

*Results of a per lesion analysis.

Surgeons, American College of Radiology, the National Cancer Institute, the US Preventive Services Task Force and the Canadian Task Force on the Periodic Health Examination all decline to recommend lung cancer screening with any test at any frequency" [9]. A recent critical review of 10 prospective trials on screening for lung cancer by Strauss *et al.* including four randomized controlled trials, two non-randomized controlled trials and four uncontrolled trials failed to show a reduction in lung cancer mortality between the experimental and control populations in the six controlled trials [10].

The efficacy of screening for second lung cancer primaries in the subset of high-risk patients with a history of prior NSCLC is unknown. In addition, technological advances in both the diagnosis and the treatment of early tracheobronchial mucosal lesions render the conclusions drawn from prior trials of historical significance only.

In March 1998 Lam *et al.* [7] published data from the multi-institutional clinical study that showed that the LIFE-Lung system, when used as an adjunct to conventional WLB, improved the physician's ability to identify moderate/severe dysplasia or worse. In this trial the efficacy of fluorescence bronchoscopy in patients with suspected or proven NSCLC was assessed: 154 of the 173 patients in this trial were suspected of having or known to have lung cancer based on symptoms and/or abnormal chest radiograph (114 patients), abnormal sputum cytology (29 patients) and 11 patients were known to have lung cancer based on prior investigations. The relative sensitivity of LIFE versus WLB for invasive carcinoma was only 1.46 (95% CI, 1.21–1.95) in this trial. However, LIFE-Lung's superiority in detecting early, intraepithelial lesions was evident in this trial. One hundred and two intraepithelial neoplasias were identified with 57 lesions diagnosed on the LIFE-Lung examination versus only nine lesions by WLB alone. Thus, the relative sensitivity for LIFE versus WLB for intraepithelial neoplasias in this trial was 6.3 (95% CI, 3.6–12.3).

At UPMC, we are utilizing the LIFE-Lung system as a surveillance tool in post-resection patients who have had a curative resection or definitive PDT for a NSCLC and are believed to be disease-free. The risk of developing second lung primaries for patients who have undergone potentially curative resections of a NSCLC is 1–3% per patient per year. The North American Lung Cancer Study Group (NLCSG) documented a 3.6% per patient per year risk of developing local recurrence and/or second lung primaries in patients with NSCLC [11]. Data from the Mayo Clinic on patients that underwent surgical resection for sputum cytology positive, roentenographically occult, lung cancers suggested that second primary lung cancers occurred at a rate as high as 5% per patient per year [12]. A recent collective review of 1406 patients with occult or stage I completely resected lung carcinomas reported an incidence of second primary lung cancers of 11.4% (range 3–30%) [1].

Patients who develop second primaries and/or local recurrence have limited curative treatment options because of their diminished pulmonary reserve as a consequence of their prior resection [13]. The peri-operative mortality for second primary lung carcinomas is significantly higher than for the first tumor and surgical options are both more limited and more complicated as a consequence of the re-operative nature of a second surgical approach [13]. In January 1998, however, the Federal Drug Administration approved PDT as a curative treatment modality for early (T1N0), NSCLCs in patients that were poor candidates for surgery and/or radiation therapy. Cortese *et al.* treated 21 patients with 23 early NSCLCs with curative intent and documented a greater than 90% complete response rate to PDT. In this series, 43% of the patients were spared surgery at 68 months follow-up [12]. The LIFE-Lung system potentially affords the physician the ability to detect, with a higher sensitivity, early stage lesions that may be amenable to definitive PDT. To date, we have treated eight lesions with PDT with curtive intent.

We are currently performing a LIFE exam to delineate the exact site of the previously treated lesion. However, in our series 25 of the biopsies at the prior PDT treatment site were false positive on LIFE exam (Table III). We speculate that this may be secondary to retained photofrin in these patients (Fig. 4). We are routinely performing LIFE

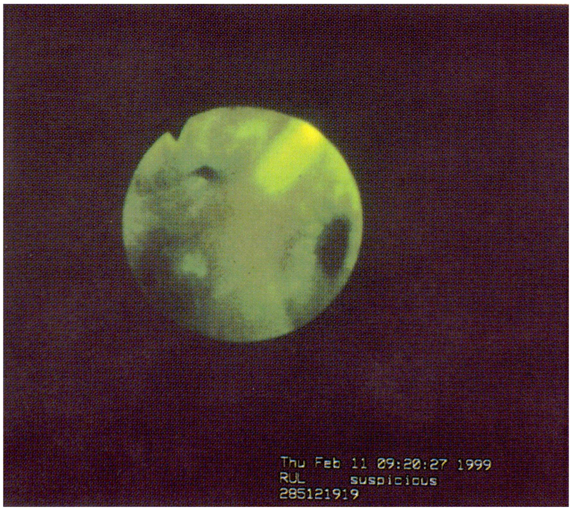

FIGURE 4 Class III LIFE image of the trifurcation of right upper lobe subsegments in a post-PDT patient. The reddish-brown area was biopsy negative.

at one and three months post-PDT to re-biopsy the former lesion to monitor for recurrent or persistent disease.

### CONCLUSIONS AND FUTURE DIRECTIONS

Early detection and careful mapping of pre-invasive lesions with the Xillix LIFE-Lung system may translate into improved survival of high-risk patients such as post-resection and post-curative PDT patients. The LIFE-Lung system can identify lesions at an early stage, when they are potentially curable with minimally invasive, therapeautic interventions such as PDT. In addition, longitudinal monitoring of IENs and PDT treated lesions with LIFE bronchoscopy may help to better define their natural history.

The current paradigm of lung carcinogenesis depicts a multistage process with potential genetic markers at each stage [14]. LIFE bonchoscopy affords investigators the opportunity to identify early mucosal abnormalities prior to the phenotypic expression of a transformed cell, i.e. before the development of an invasive carcinoma. Biopsy specimens from these pre-malignant lesions may help unravel the cascade of molecular events that occur in tracheobronchial mucosa carcinogenesis. Identification of early molecular markers may facilitate the development of efficient, molecular sputum screening techniques for NSCLC as an adjunct to, and/or replacement for traditional, costly, labor-intense cytologic screening methods.

The Xillix® LIFE-Lung Fluorescent Endoscopy System is a registered trademark of Xillix Technologies Corporation of Richmond, B.C., Canada.

### References

[1] Lam, S. and Becker, H.D. Future diagnostic procedures. *Chest Surgery Clinics of North America* 1996; **6**(2): 363–380.
[2] Auerbach, O., Stout, A.P., Hammond, E.C. *et al.* Changes in bronchial epithelium in relation to cigarette smoking and in relation to lung cancer. *N. Engl. J. Med.* 1961; **265**(6): 253–267.
[3] Saccomanno, G., Archer, V.E., Auerbach, O. *et al.* Development of Carcinoma of the Lungs Reflected in Exfoliated Cells. *Cancer* 1974; **33**(1): 256–270.
[4] Woolner, L.B., Fontana, R.S., Cortese, D.A. *et al.* Roentgenographically occult lung cancer: Pathologic findings and frequency of multicentricity during a 10-year period. *Mayo Clin. Proceedings* 1984; **59**(7): 453–466.
[5] Palcic, B., Lam, S., Hung, J. *et al.* Detection and localization of early lung cancer by imaging techniques. *Chest* 1991; **99**(3): 742–743.
[6] Lam, S. and Palcic, B. Flourescence detection. In: Roth, J.A. *et al.* (Eds). *Lung Cancer.* Boston: Blackwell Scientific Publications, 1991; pp. 325–338.
[7] Lam, S., Kennedy, T., Unger, M. *et al.* Localization of bronchial intracpithelial neoplastic lesions by fluorescence bronchoscopy. *Chest* 1998; **113**(3): 696–702.
[8] Strauss, G.M. Measuring effectiveness of lung cancer screening: From consensus to controversy and back. *Chest* 1997; **112**(4 Suppl.): 213S–228S.
[9] Eddy, D. Screening for lung cancer. *Ann. Intern. Med.* 1989; **111**: 232–237.
[10] Strauss, G.M., Glcasen, R.E. and Sugarbaker, D.J. Screening for lung cancer: Another look; a different view. *Chest* 1997; **111**(3): 754–768.
[11] Thomas, P., Rubinstein, L. and the Lung Cancer Study Group. Cancer recurrence after resection: T1N0 non-small cell lung cancer. *Ann. Thorac. Surg.* 1990; **49**(2): 243.
[12] Cortese, D.A., Pairolero, P.C., Bergstralh, E.J. *et al.* Roentgenographically occult lung cancer: A ten year experience. *J. Thorac. Cardiovasc. Surg.* 1983; **86**(3): 373–380.
[13] Pairolero, D.C., Williams, D.E., Bergstralh, E.J. *et al.* Post surgical stage I bronchogenic carcinoma: Morbid implications of recurrent disease. *Ann. Thorac. Surg.* 1984; **38**(4): 331–338.
[14] Tockman, M.S., Gupta, P.K., Pressman, N.J. *et al.* Considerations in bringing a cnacer biomarker to clinical application. *Cancer Research* 1992; **52**(9 Suppl.): 2711s–2718s.

